# Supplementary material for: Generalizing predictions to unseen sequencing profiles via deep generative models
Source: Sci Rep. 2022 May 3;12:7151. doi: 10.1038/s41598-022-11363-w (PMC9065080; doi:10.1038/s41598-022-11363-w)
Supplement: Supplementary file 1 — Supplementary Information. [file 41598_2022_11363_MOESM1_ESM.pdf]

Supplementary materials of

Generalizing predictions to unseen sequencing profiles via deep generative modeling

Min Oh[1] and Liqing Zhang[1, *]

[1]Department of Computer Science, Virginia Tech, Blacksburg, VA, USA

[*]Correspondence: lqzhang@cs.vt.edu

Supplementary figures:

- **Figure S1.** Visualization of tumor gene expression profiles of melanoma patients.
- **Figure S2.** Visualization of microbiome marker profiles of diabetic patients and healthy controls.
- **Figure S3.** Prediction performance on unseen data (AUPRC).
- **Figure S4.** Prediction performance on unseen data with Random forest (RF) classifier.
- **Figure S5.** Anti-PD1 therapy response prediction performance (AUROC) on unseen data by varying the number of visual clusters and that of GAN models.
- **Figure S6.** Anti-PD1 therapy response prediction performance (AUPRC) on unseen data by varying the number of visual clusters and that of GAN models.
- **Figure S7.** Type 2 diabetes prediction performance (AUROC) on unseen data by varying the number of visual clusters and that of GAN models.
- **Figure S8.** Type 2 diabetes prediction performance (AUPRC) on unseen data by varying the number of visual clusters and that of GAN models.
- **Figure S9.** t-SNE visualization of augmented tumor expression profiles derived from Random augmentation along with the source and test (unseen) data of melanoma patients treated with anti-PD1 therapy.
- **Figure S10.** t-SNE visualization of augmented tumor expression profiles derived from GMM along with the source and test (unseen) data of melanoma patients treated with anti-PD1 therapy.
- **Figure S11.** t-SNE visualization of augmented tumor expression profiles derived from SMOTE along with the source and test (unseen) data of melanoma patients treated with anti-PD1 therapy.
- **Figure S12.** t-SNE visualization of augmented microbiome profiles derived from DeepBioGen along with the source and test (unseen) data of diabetic patients and healthy controls.
- **Figure S13.** t-SNE visualization of augmented microbiome profiles derived from Random augmentation along with the source and test (unseen) data of diabetic patients and healthy controls.
- **Figure S14.** t-SNE visualization of augmented microbiome profiles derived from GMM along with the source and test (unseen) data of diabetic patients and healthy controls.
- **Figure S15.** t-SNE visualization of augmented microbiome profiles derived from SMOTE along with the source and test (unseen) data of diabetic patients and healthy controls.
- **Figure S16.** Feature-wise WSS by the number of clusters.
- **Figure S17.** Conditional Wasserstein GAN architecture in DeepBioGen.
- **Figure S18.** Sample-wise WSS by the number of clusters.

Supplementary Tables:

- **Table S1.** Summary of sequencing data sets.
- **Table S2.** Hyper-parameter grid for optimizing classifiers.
- **Table S3.** Modified inception scores of generated sequencing profiles varying the number of conditional Wasserstein GANs.
- **Table S4.** Anti-PD1 therapy responsiveness prediction accuracy of the best classifiers on unseen data.
- **Table S5.** Type 2 diabetes prediction accuracy of the best classifiers on unseen data.
- **Table S6.** 5-fold cross-validation on 126 tumor expression profiles (source data only).
- **Table S7.** 5-fold cross-validation on 344 gut microbiome profiles (source data only).
- **Table S8.** 5-fold cross-validation on 176 tumor expression profiles (source + test data).
- **Table S9.** 5-fold cross-validation on 440 gut microbiome profiles (source + test data).

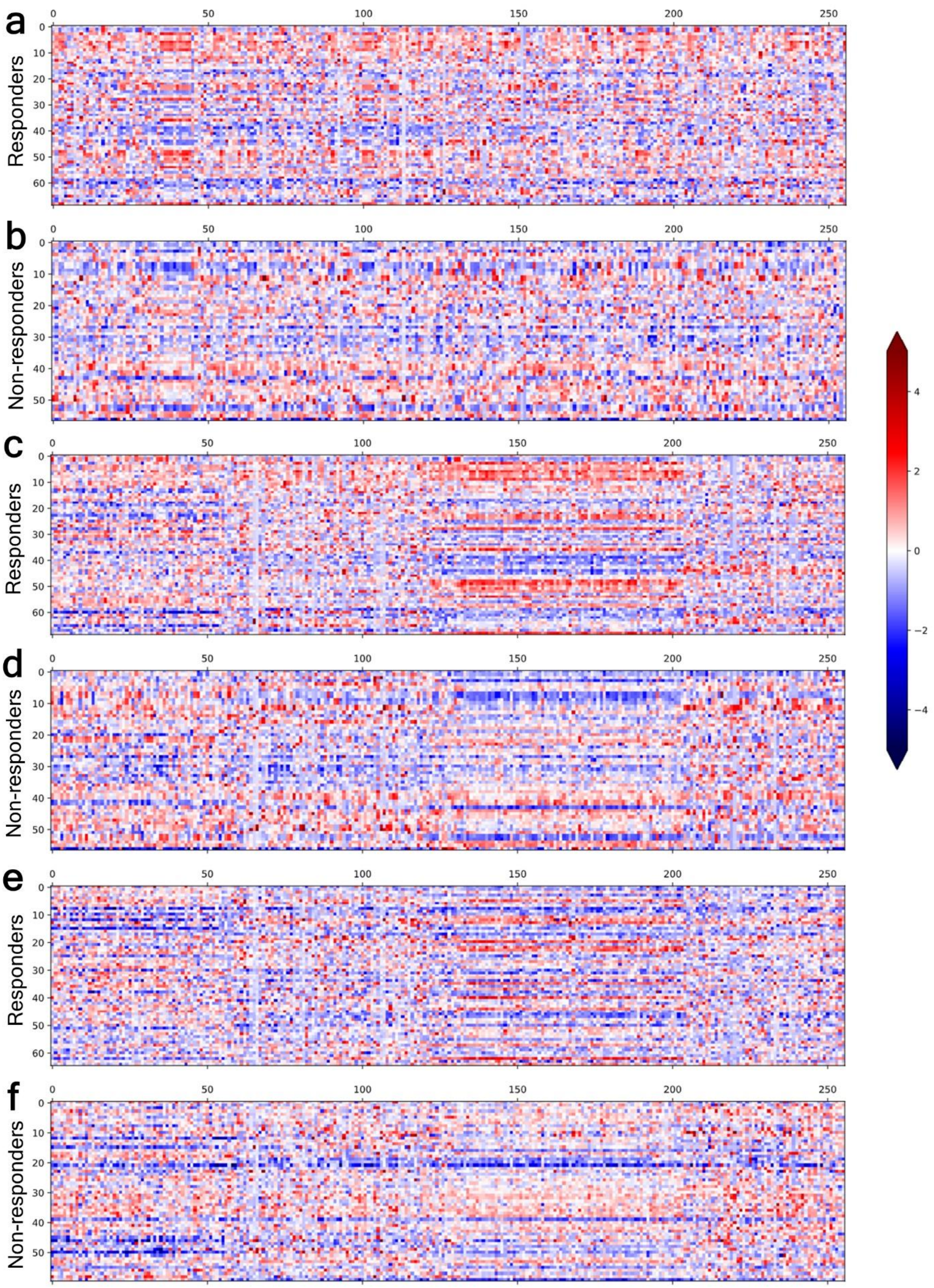

Figure S1. Visualization of tumor gene expression profiles of melanoma patients. **a-b**, The columns are

unordered genes before pre-processing, and each row indicates the profile of responder (a) or non-responder (b). **c-d**, The columns are re-ordered genes with 4 clusters derived from feature-wise clustering. **e-f**, The augmented profiles generated by DeepBioGen.

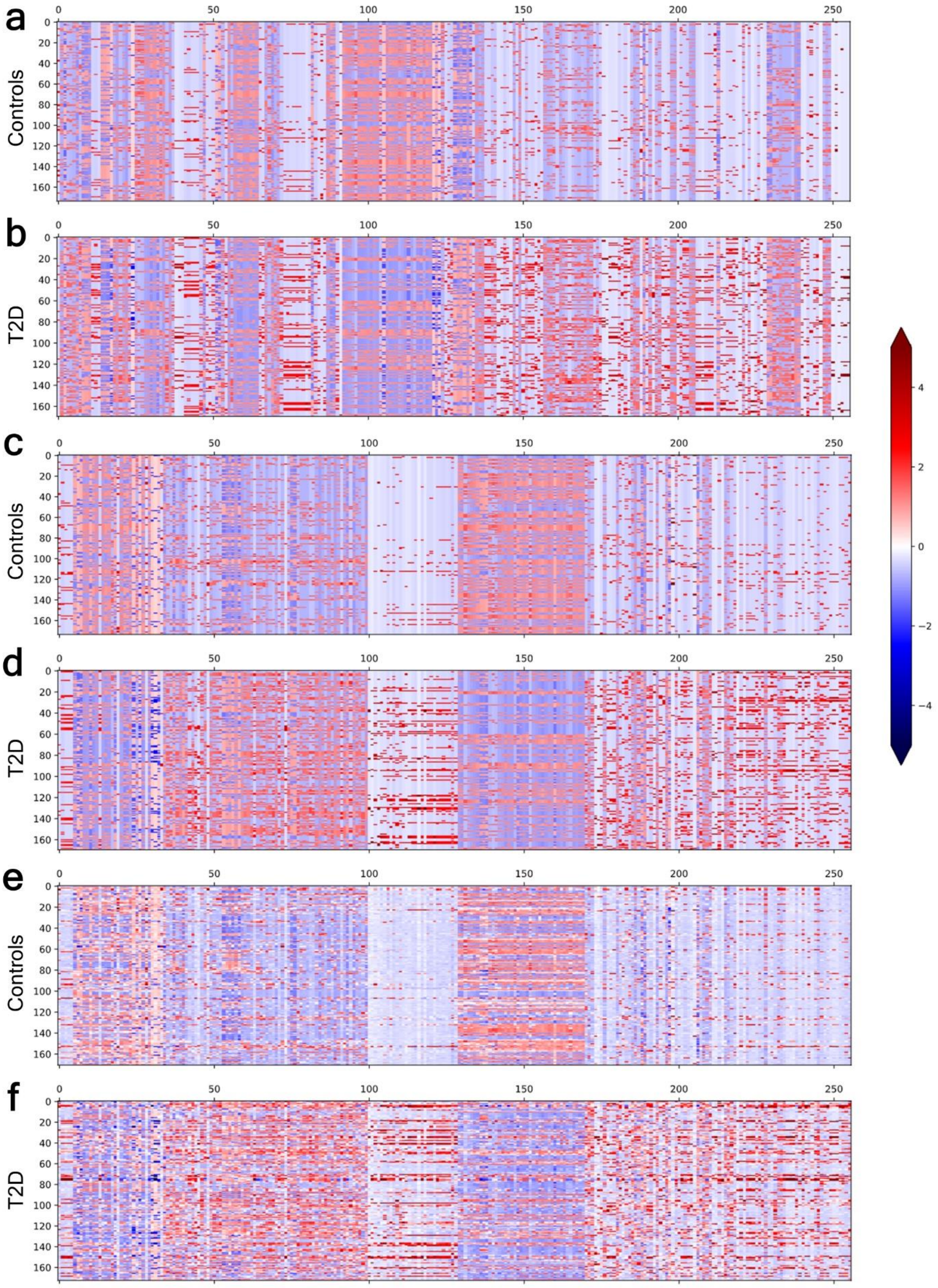

Figure S2. Visualization of microbiome marker profiles of diabetic patients and healthy controls. **a-b**,

The columns are unordered genes before pre-processing, and each row indicates the profile of healthy controls (a) or type 2 diabetes (b). **c-d**, The columns are re-ordered genes with 4 clusters derived from feature-wise clustering. **e-f**, The augmented profiles generated by DeepBioGen.

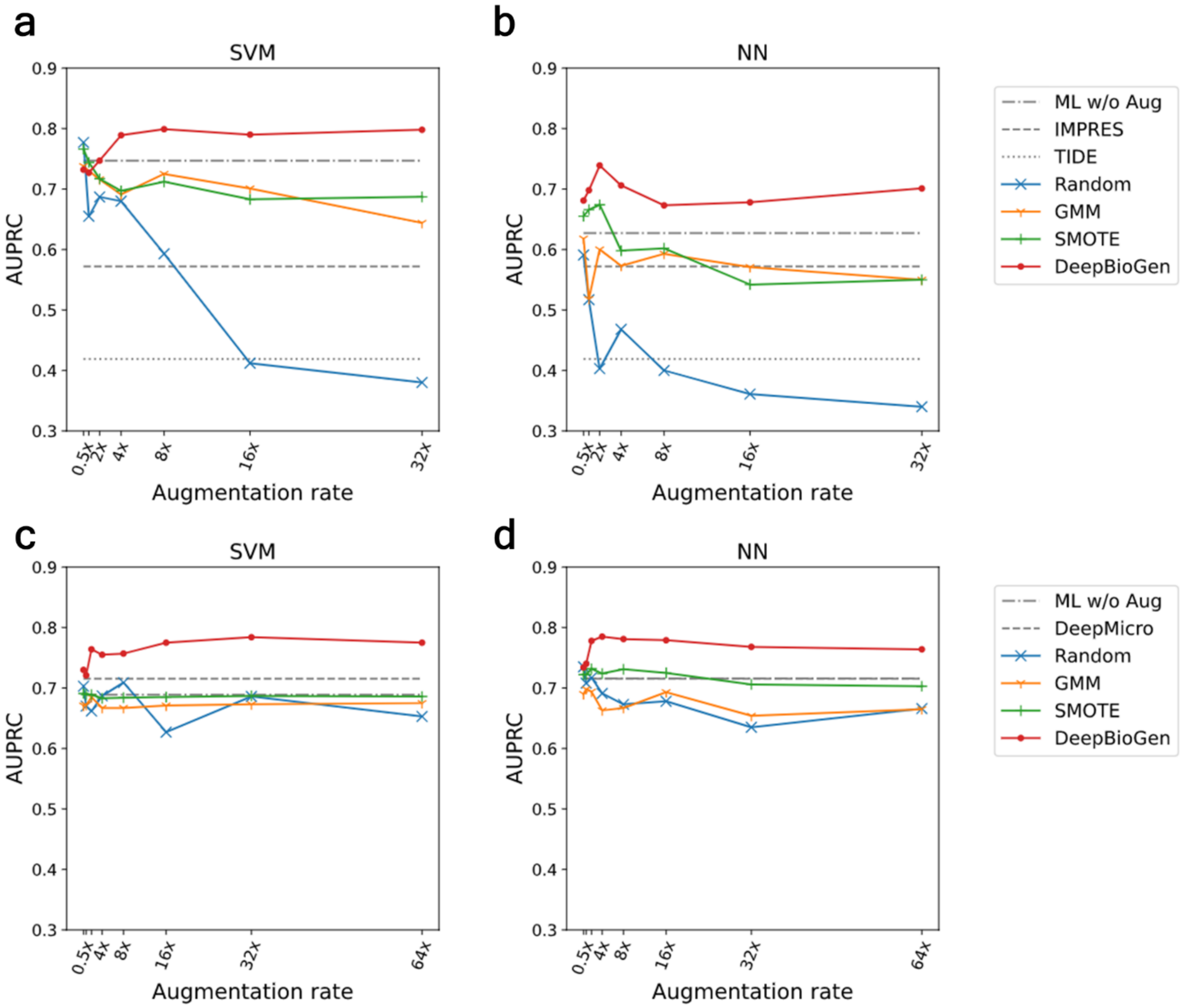

Figure S3. Prediction performance on unseen data (AUPRC). **a-b**, Results of anti-PD1 therapy response prediction on unseen data by the state-or-the-art and baseline classifiers (gray) and by classifiers generalized with DeepBioGen (red), SMOTE (green), GMM (yellow), and Random augmentation (blue); Classification algorithms: Support Vector Machine (SVM) and Neural network (NN) which is a multi-layer perceptron; Evaluation metric: Area under the precision-recall curve (AUPRC). **c-d**, Results of type 2 diabetes prediction on unseen data.

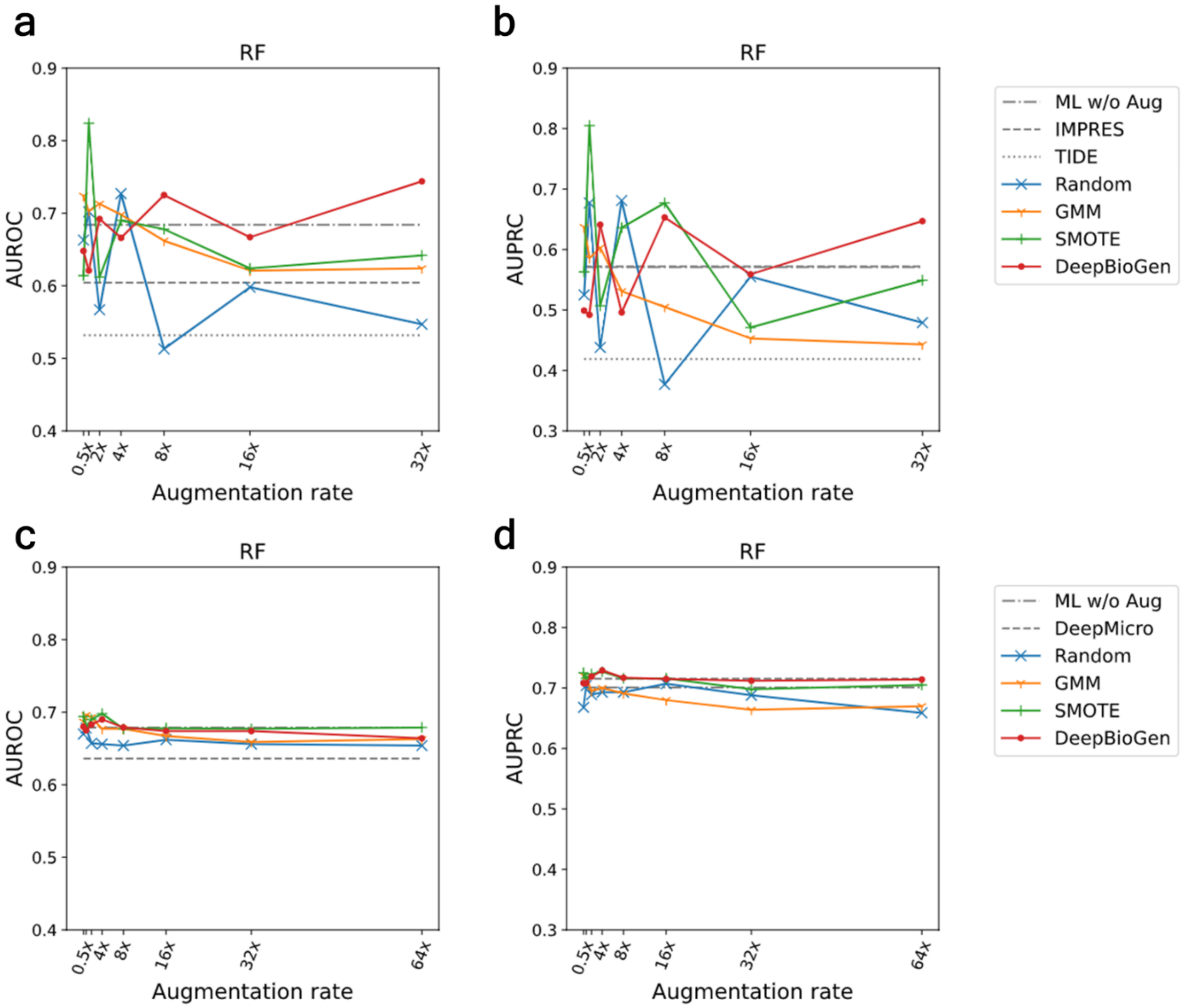

Figure S4. Prediction performance on unseen data with Random forest (RF) classifier. **a-b**, Results of anti-PD1 therapy response prediction on unseen data by the state-or-the-art and baseline classifiers (gray) and by the classifier generalized with DeepBioGen (red), SMOTE (green), GMM (yellow), and Random augmentation (blue); Evaluation metrics: Area under the receiver operating characteristics (AUROC) and Area under the precision-recall curve (AUPRC). **c-d**, Results of type 2 diabetes prediction on unseen data; Evaluation metric: AUROC and AUPRC.

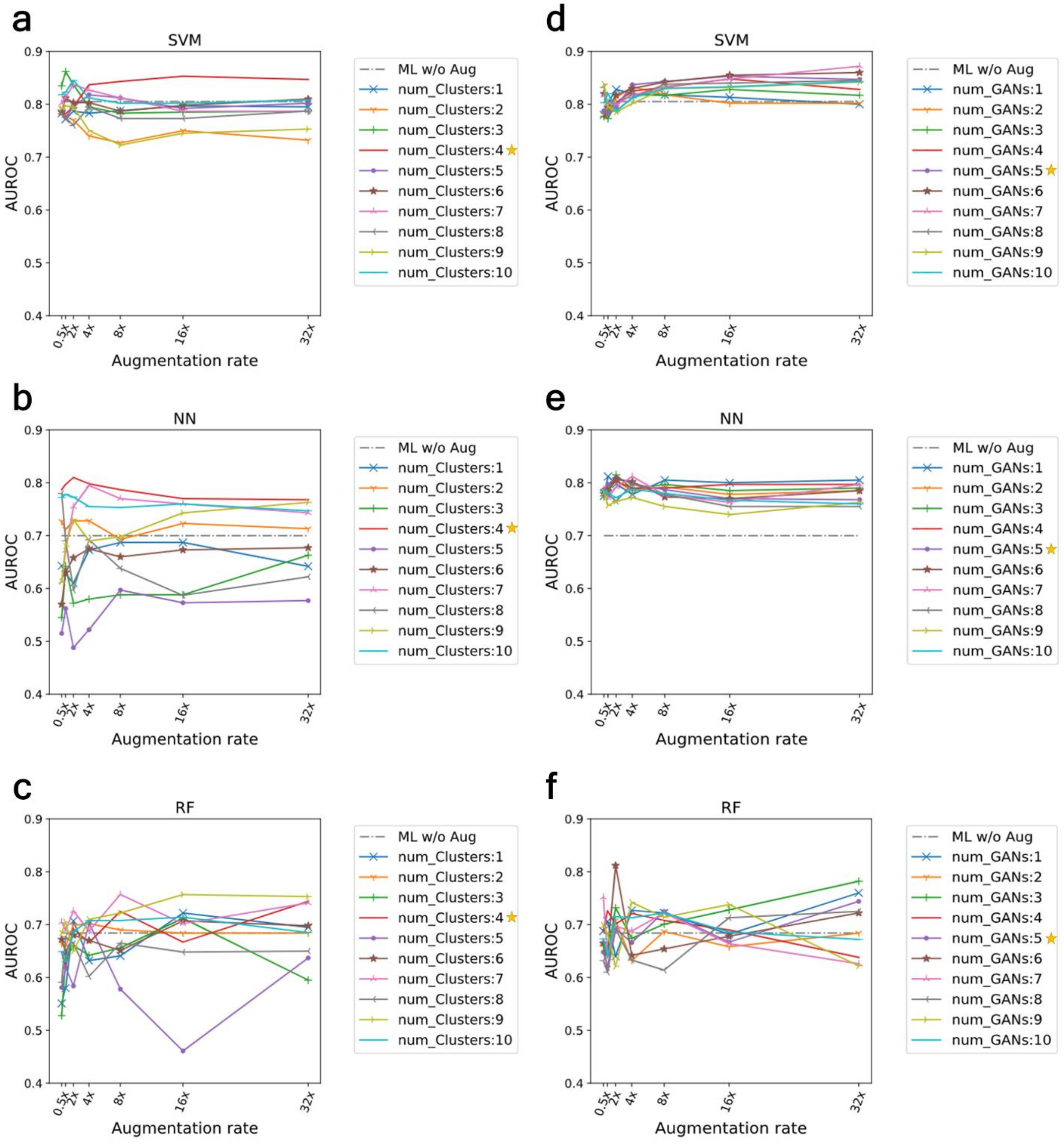

Figure S5. Anti-PD1 therapy response prediction performance (AUROC) on unseen data by varying the number of visual clusters and that of GAN models. **a-c**, Varying the number of visual clusters while fixing the number of GANs as five; Yellow star denotes the estimated number of visual clusters. **d-f**, Varying the number of GANs while fixing the number of visual clusters as four; Yellow start denotes the estimated number of GANs.

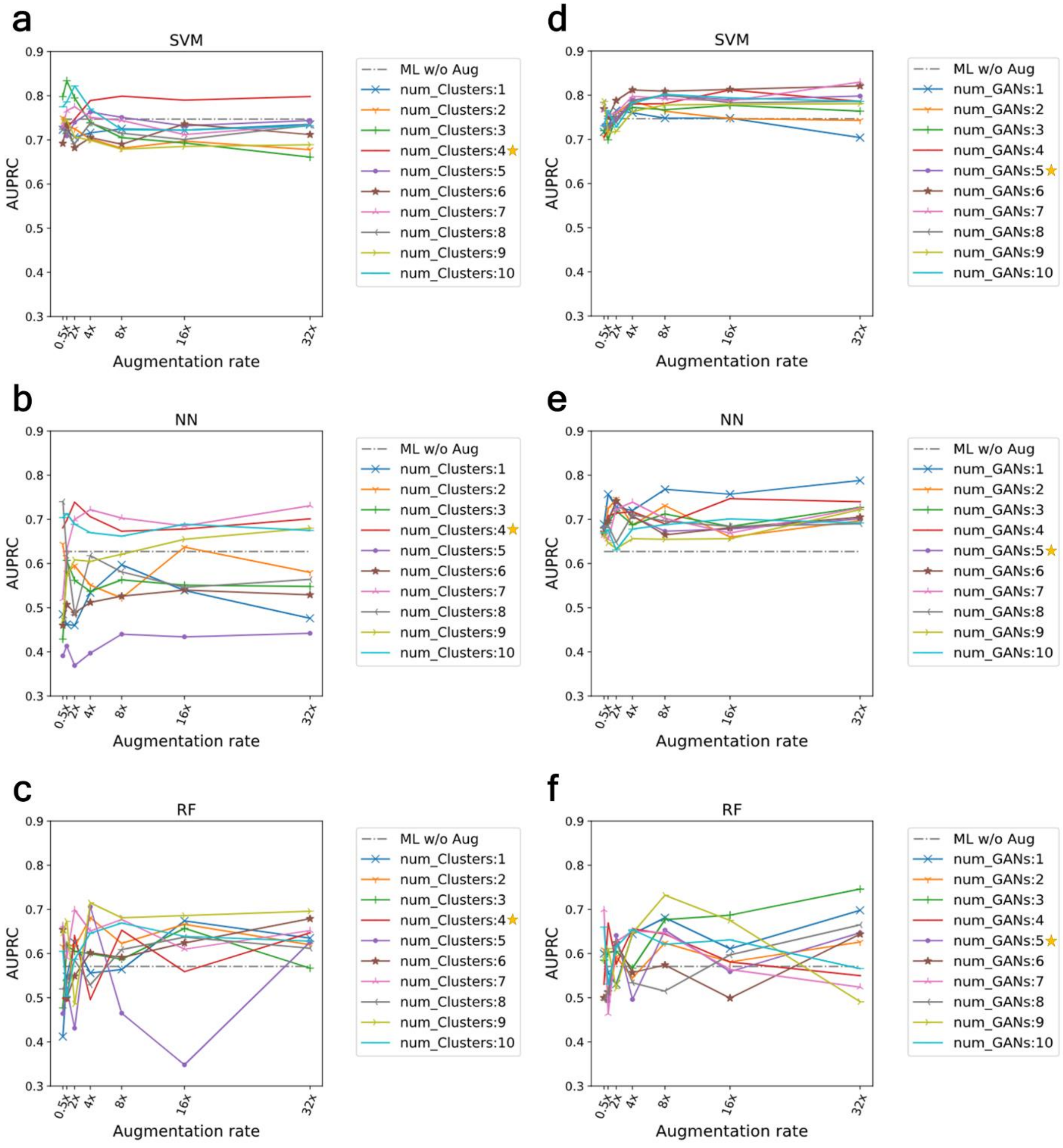

Figure S6. Anti-PD1 therapy response prediction performance (AUPRC) on unseen data by varying the number of visual clusters and that of GAN models. **a-c**, Varying the number of visual clusters while fixing the number of GANs as five; Yellow star denotes the estimated number of visual clusters. **d-f**, Varying the number of GANs while fixing the number of visual clusters as four; Yellow start denotes the estimated number of GANs.

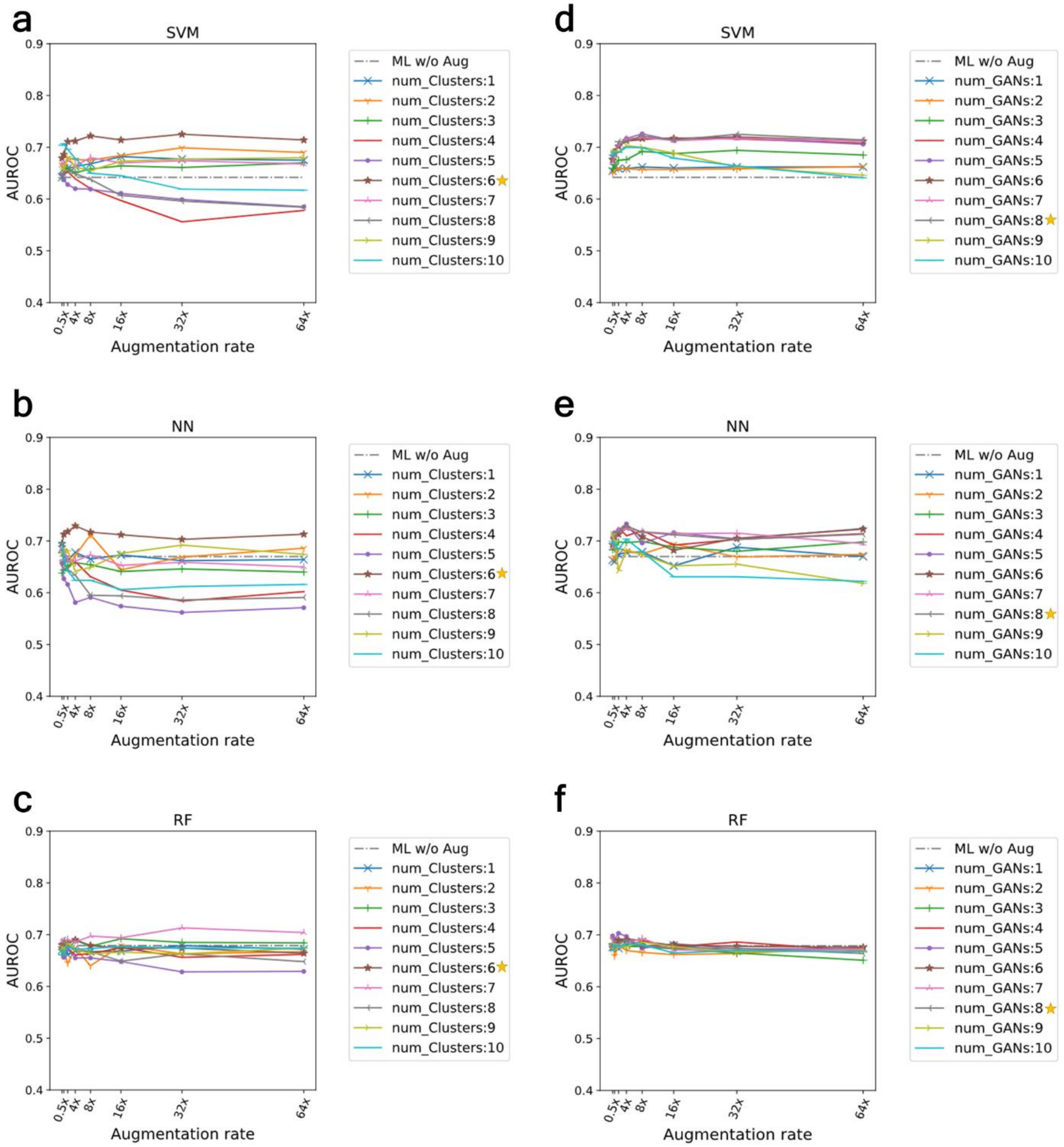

Figure S7. Type 2 diabetes prediction performance (AUROC) on unseen data by varying the number of visual clusters and that of GAN models. **a-c**, Varying the number of visual clusters while fixing the number of GANs as eight; Yellow star denotes the estimated number of visual clusters. **d-f**, Varying the number of GANs while fixing the number of visual clusters as six; Yellow start denotes the estimated number of GANs.

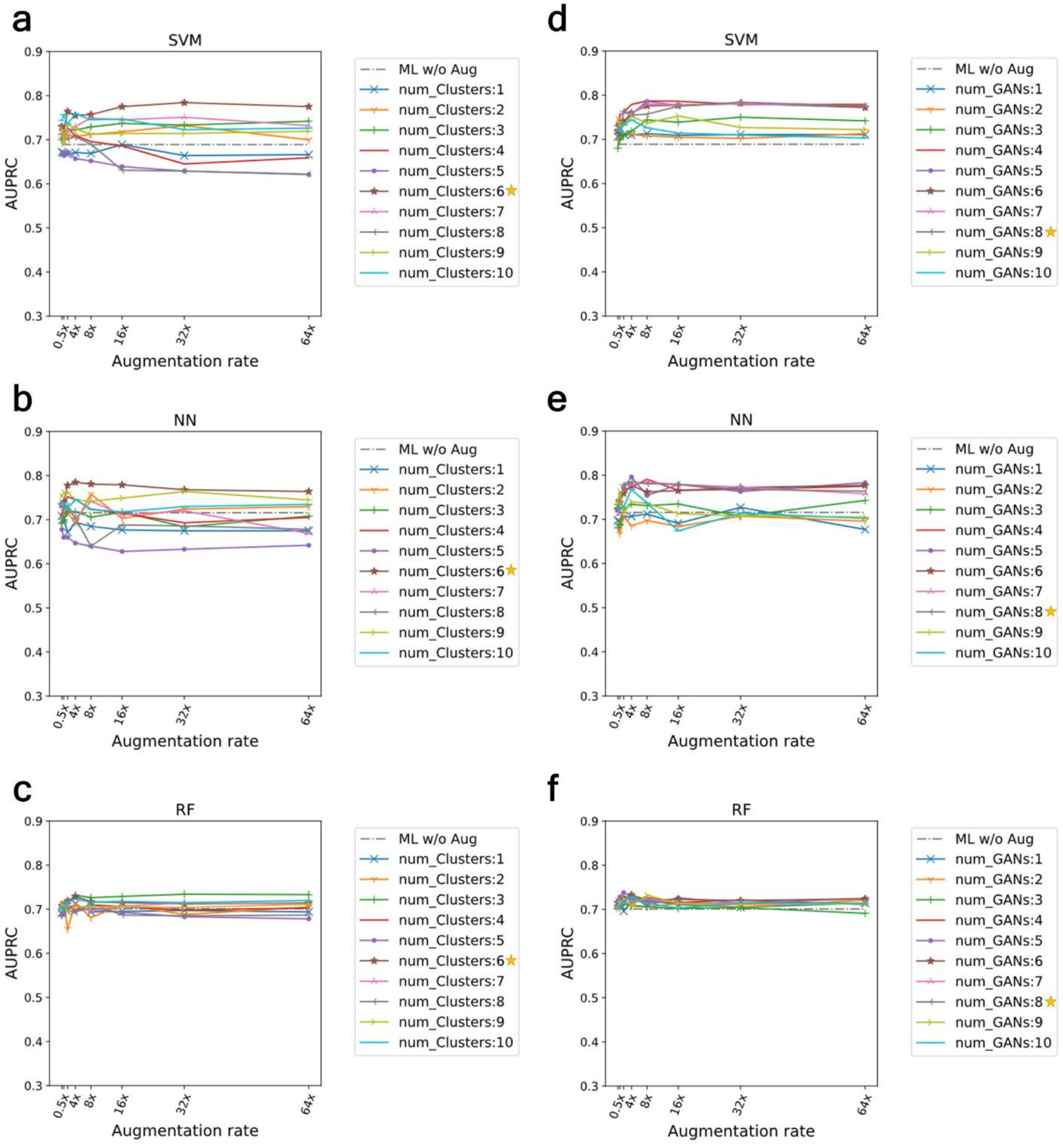

Figure S8. Type 2 diabetes prediction performance (AUPRC) on unseen data by varying the number of visual clusters and that of GAN models. **a-c**, Varying the number of visual clusters while fixing the number of GANs as eight; Yellow star denotes the estimated number of visual clusters. **d-f**, Varying the number of GANs while fixing the number of visual clusters as six; Yellow start denotes the estimated number of GANs.

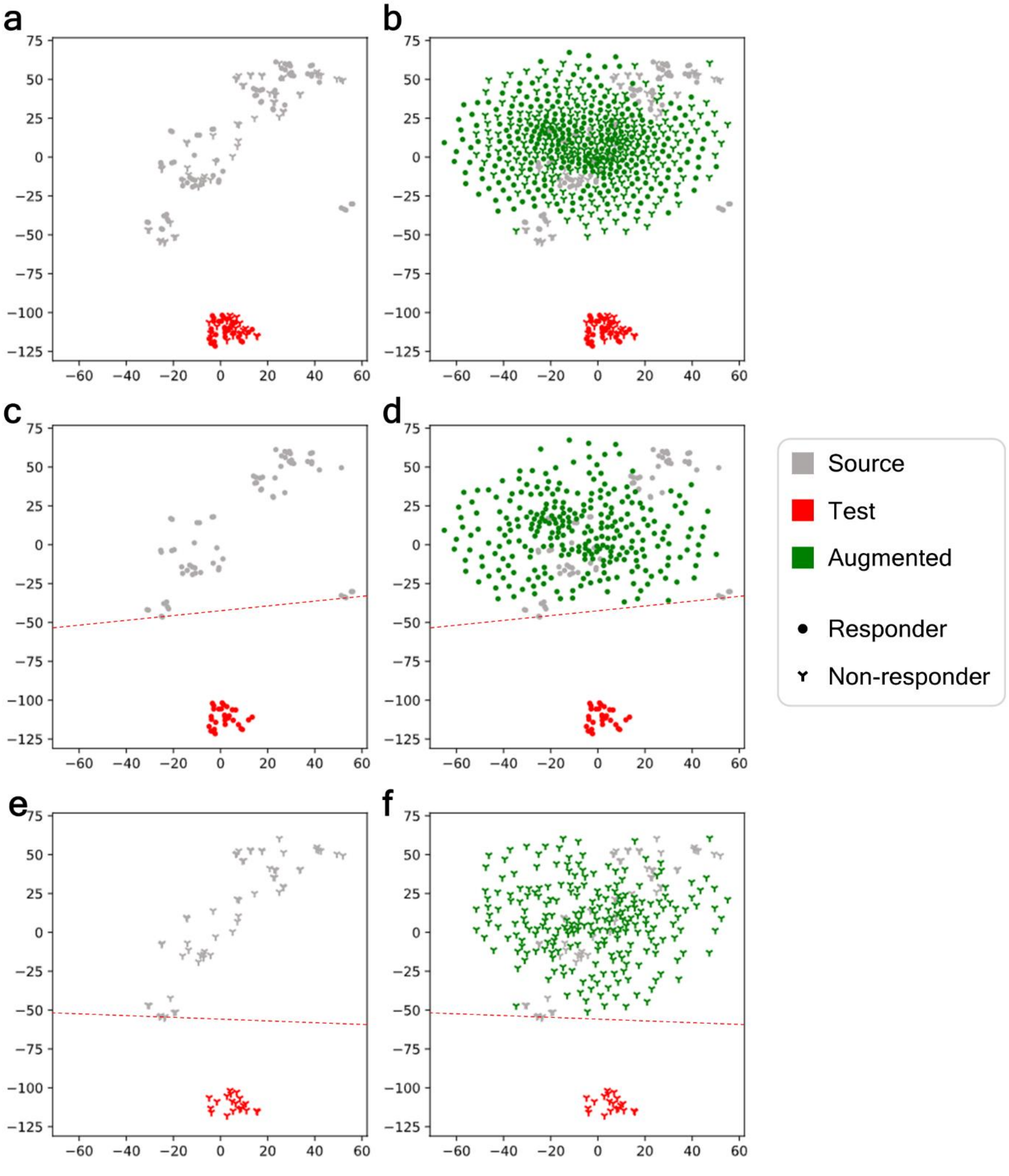

Figure S9. t-SNE visualization of augmented tumor expression profiles derived from Random augmentation along with the source and test (unseen) data of melanoma patients treated with anti-PD1 therapy. **a**, The source (gray) and test data (red). **b**, The source, test, and augmented data (green). **c**, Responders of the source and test data; An empirical boundary of responders of source data (red dotted line). **d**, Responders of the source, test, and augmented data. **e**, Non-responders of the source and test data; An empirical boundary of non-responders of source data (red dotted line). **f**, Non-responders of the source, test, and augmented data.

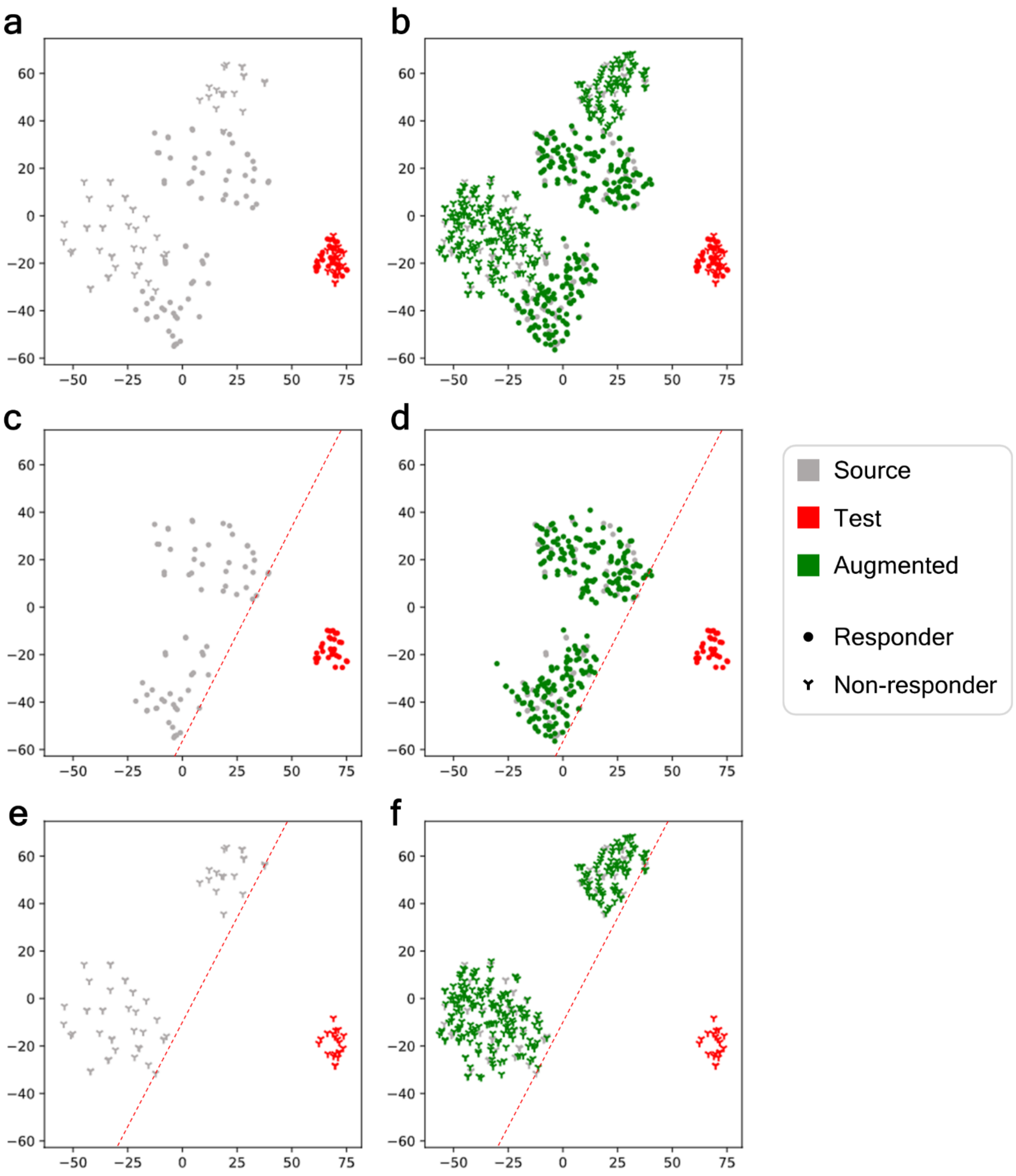

Figure S10. t-SNE visualization of augmented tumor expression profiles derived from GMM along with the source and test (unseen) data of melanoma patients treated with anti-PD1 therapy. **a**, The source (gray) and test data (red). **b**, The source, test, and augmented data (green). **c**, Responders of the source and test data; An empirical boundary of responders of source data (red dotted line). **d**, Responders of the source, test, and augmented data. **e**, Non-responders of the source and test data; An empirical boundary of non-responders of source data (red dotted line). **f**, Non-responders of the source, test, and augmented data.

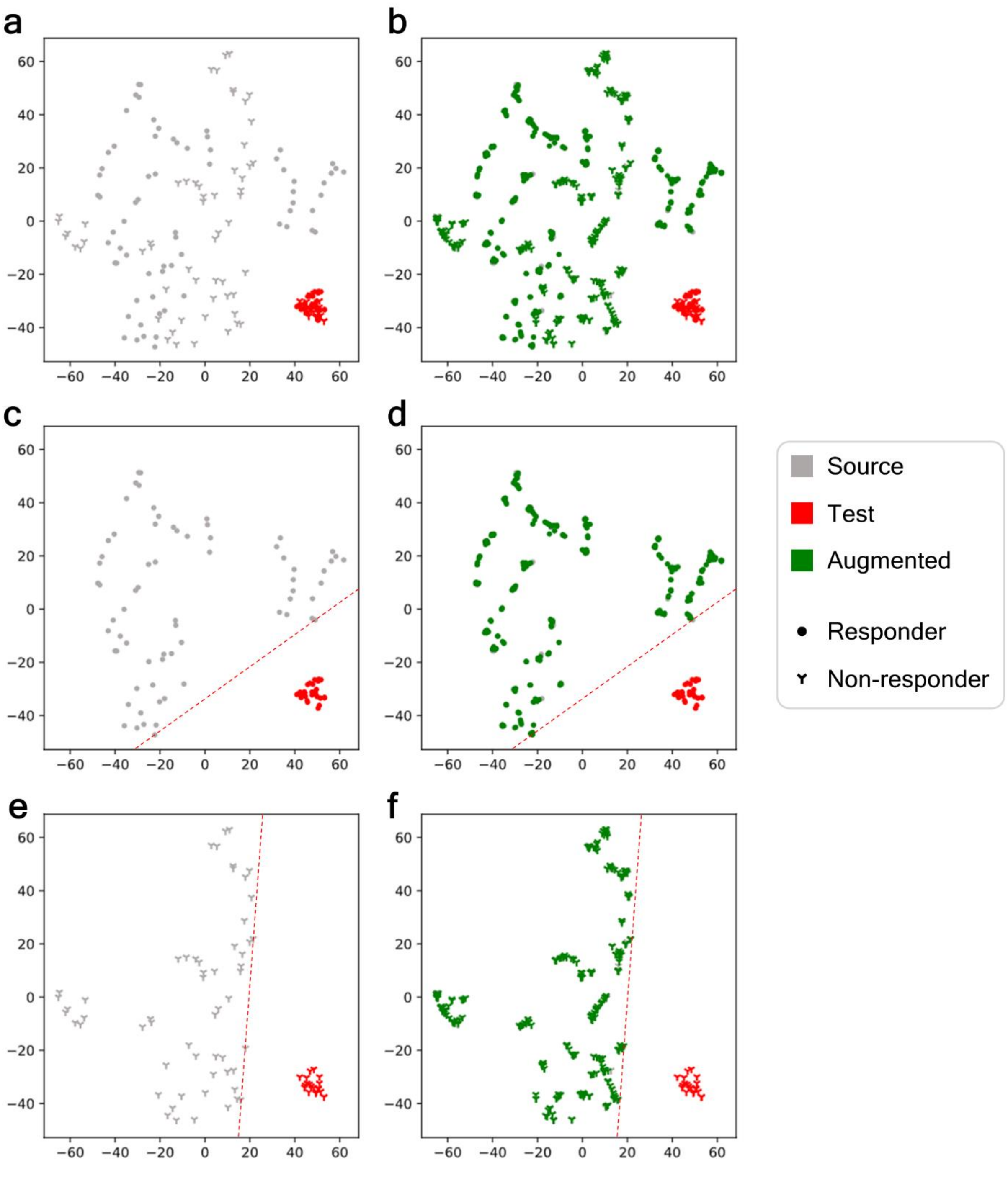

Figure S11. t-SNE visualization of augmented tumor expression profiles derived from SMOTE along with the source and test (unseen) data of melanoma patients treated with anti-PD1 therapy. **a**, The source (gray) and test data (red). **b**, The source, test, and augmented data (green). **c**, Responders of the source and test data; An empirical boundary of responders of source data (red dotted line). **d**, Responders of the source, test, and augmented data. **e**, Non-responders of the source and test data; An empirical boundary of non-responders of source data (red dotted line). **f**, Non-responders of the source, test, and augmented data.

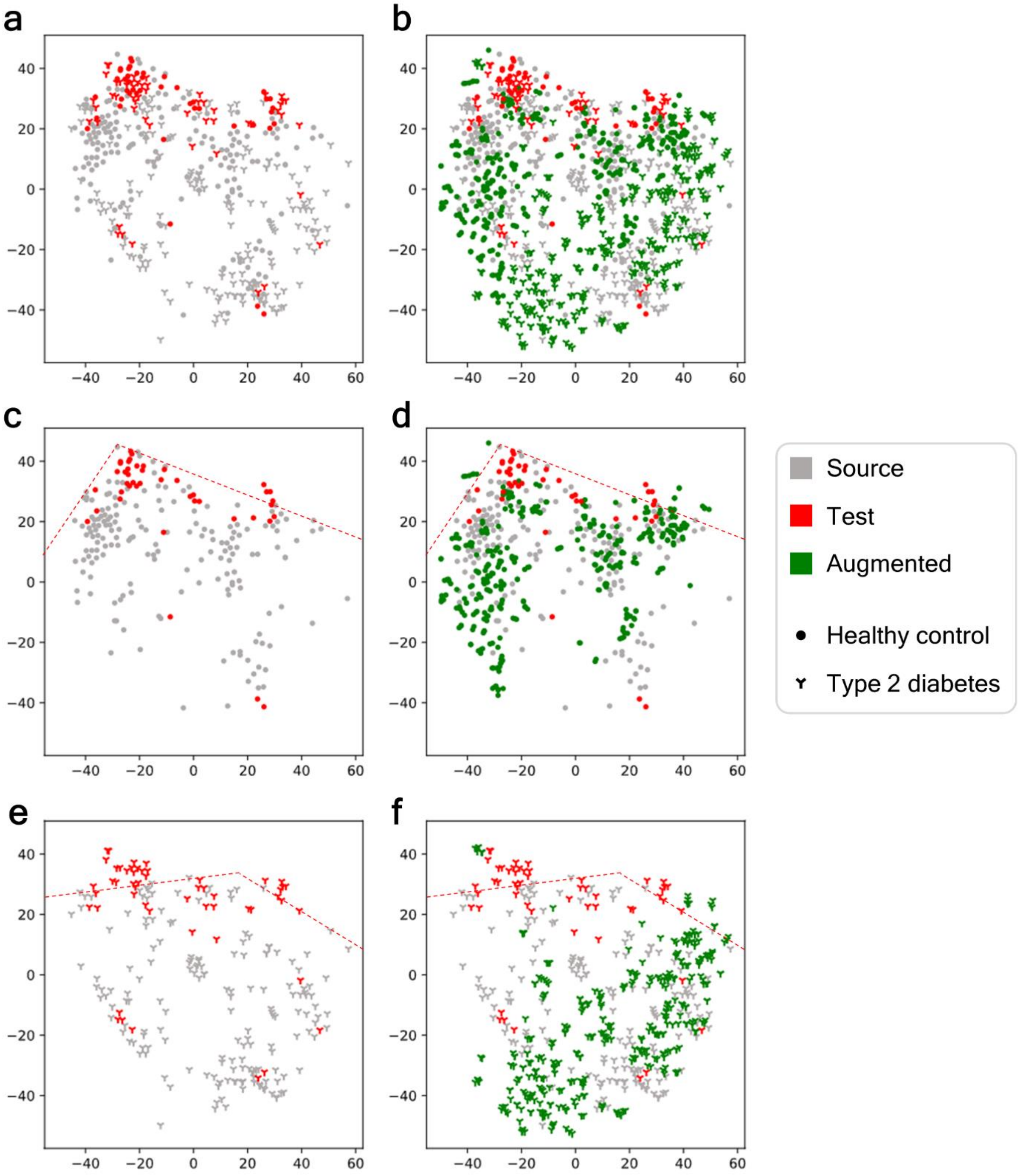

Figure S12. t-SNE visualization of augmented microbiome profiles derived from DeepBioGen along with the source and test (unseen) data of diabetic patients and healthy controls. **a**, The source (gray) and test data (red). **b**, The source, test, and augmented data (green). **c**, Healthy controls of the source and test data; An empirical boundary of healthy controls of source data (red dotted line). **d**, Healthy controls of the source, test, and augmented data. **e**, Type 2 diabetes patients of the source and test data; An empirical boundary of type 2 diabetes patients of source data (red dotted line). **f**, Type 2 diabetes patients of the source, test, and augmented data.

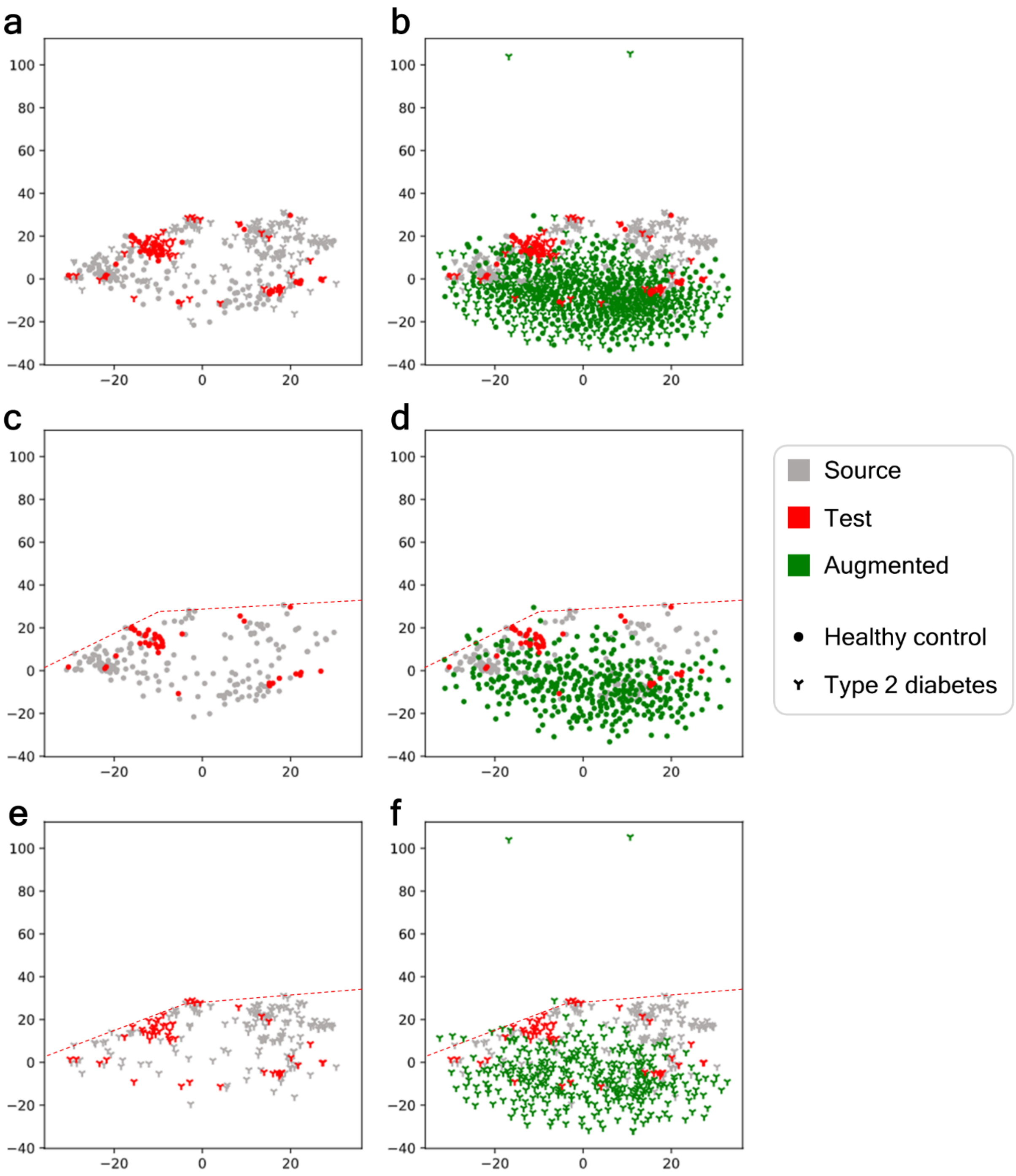

Figure S13. t-SNE visualization of augmented microbiome profiles derived from Random augmentation along with the source and test (unseen) data of diabetic patients and healthy controls. **a**, The source (gray) and test data (red). **b**, The source, test, and augmented data (green). **c**, Healthy controls of the source and test data; An empirical boundary of healthy controls of source data (red dotted line). **d**, Healthy controls of the source, test, and augmented data. **e**, Type 2 diabetes patients of the source and test data; An empirical boundary of type 2 diabetes patients of source data (red dotted line). **f**, Type 2 diabetes patients of the source, test, and augmented data.

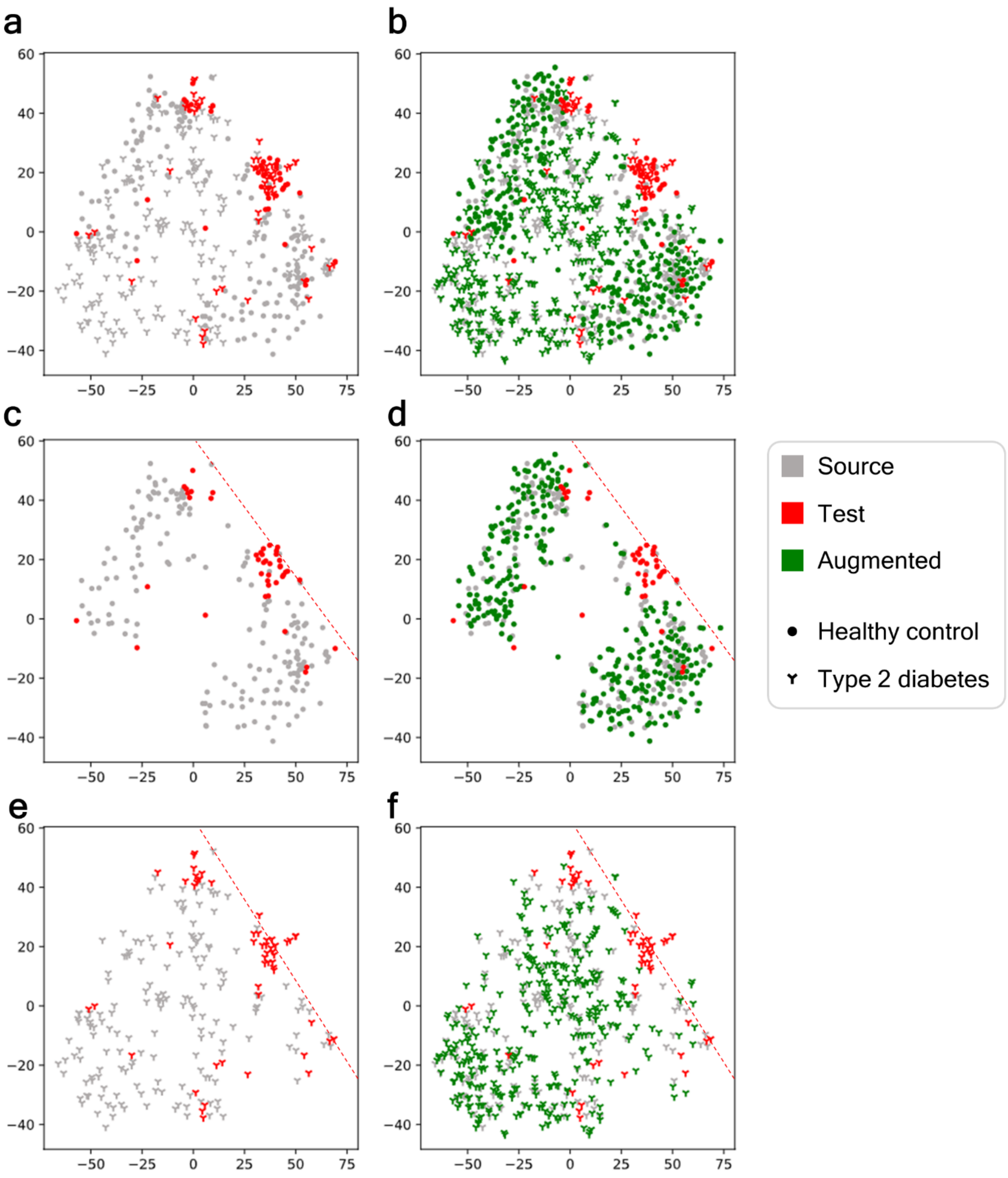

Figure S14. t-SNE visualization of augmented microbiome profiles derived from GMM along with the source and test (unseen) data of diabetic patients and healthy controls. **a**, The source (gray) and test data (red). **b**, The source, test, and augmented data (green). **c**, Healthy controls of the source and test data; An empirical boundary of healthy controls of source data (red dotted line). **d**, Healthy controls of the source, test, and augmented data. **e**, Type 2 diabetes patients of the source and test data; An empirical boundary of type 2 diabetes patients of source data (red dotted line). **f**, Type 2 diabetes patients of the source, test, and augmented data.

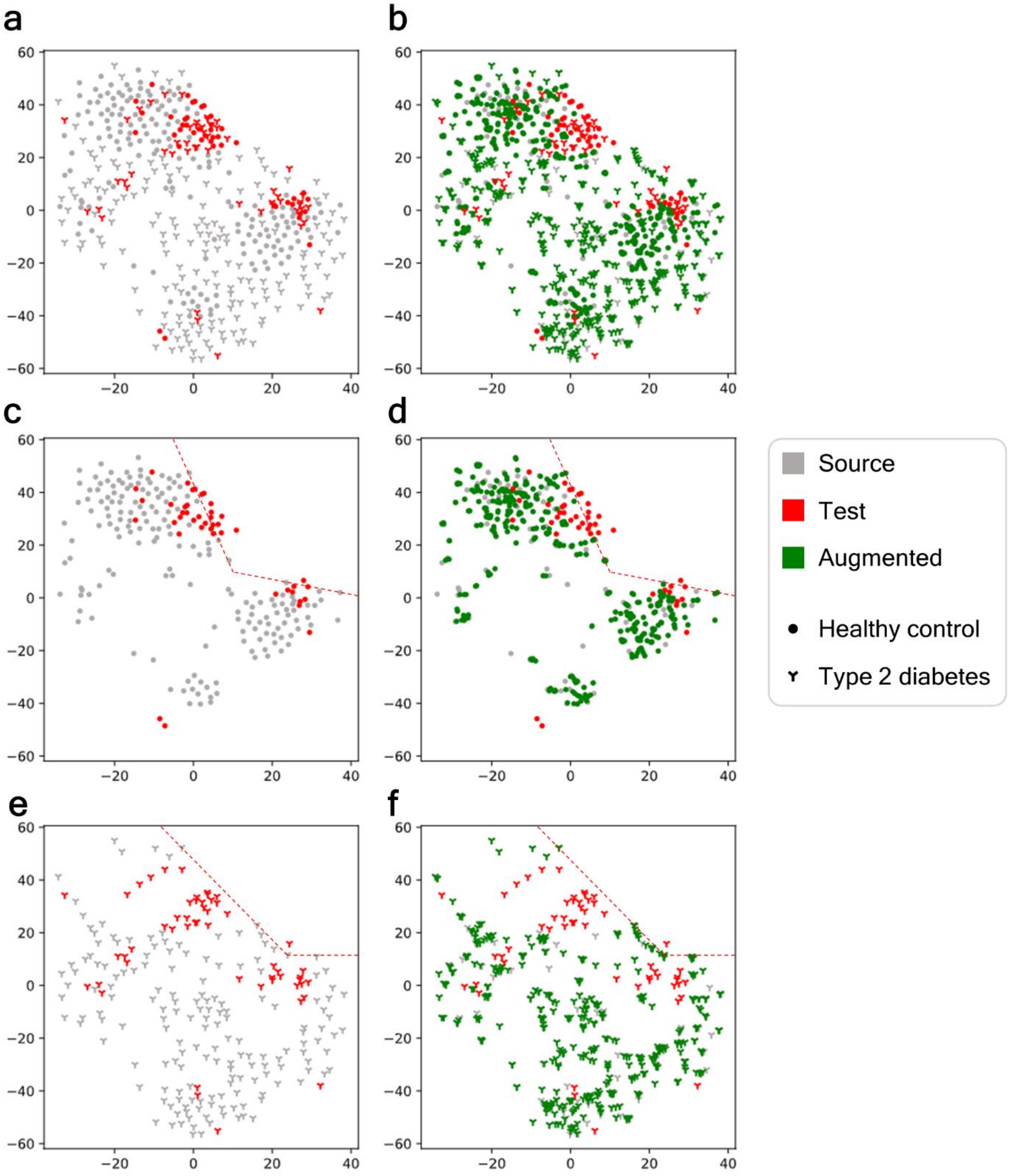

Figure S15. t-SNE visualization of augmented microbiome profiles derived from SMOTE along with the source and test (unseen) data of diabetic patients and healthy controls. **a**, The source (gray) and test data (red). **b**, The source, test, and augmented data (green). **c**, Healthy controls of the source and test data; An empirical boundary of healthy controls of source data (red dotted line). **d**, Healthy controls of the source, test, and augmented data. **e**, Type 2 diabetes patients of the source and test data; An empirical boundary of type 2 diabetes patients of source data (red dotted line). **f**, Type 2 diabetes patients of the source, test, and augmented data.

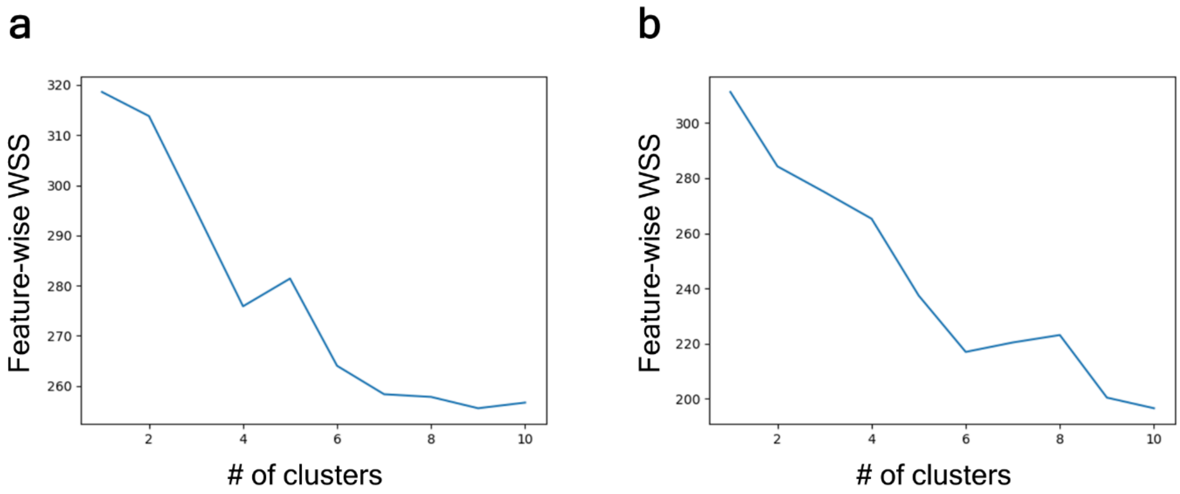

Figure S16. Feature-wise WSS by the number of clusters. **a**, RNA-seq tumor expression profiles (optimum: 4). **b**, WGS human gut microbiome marker profile (optimum: 6).

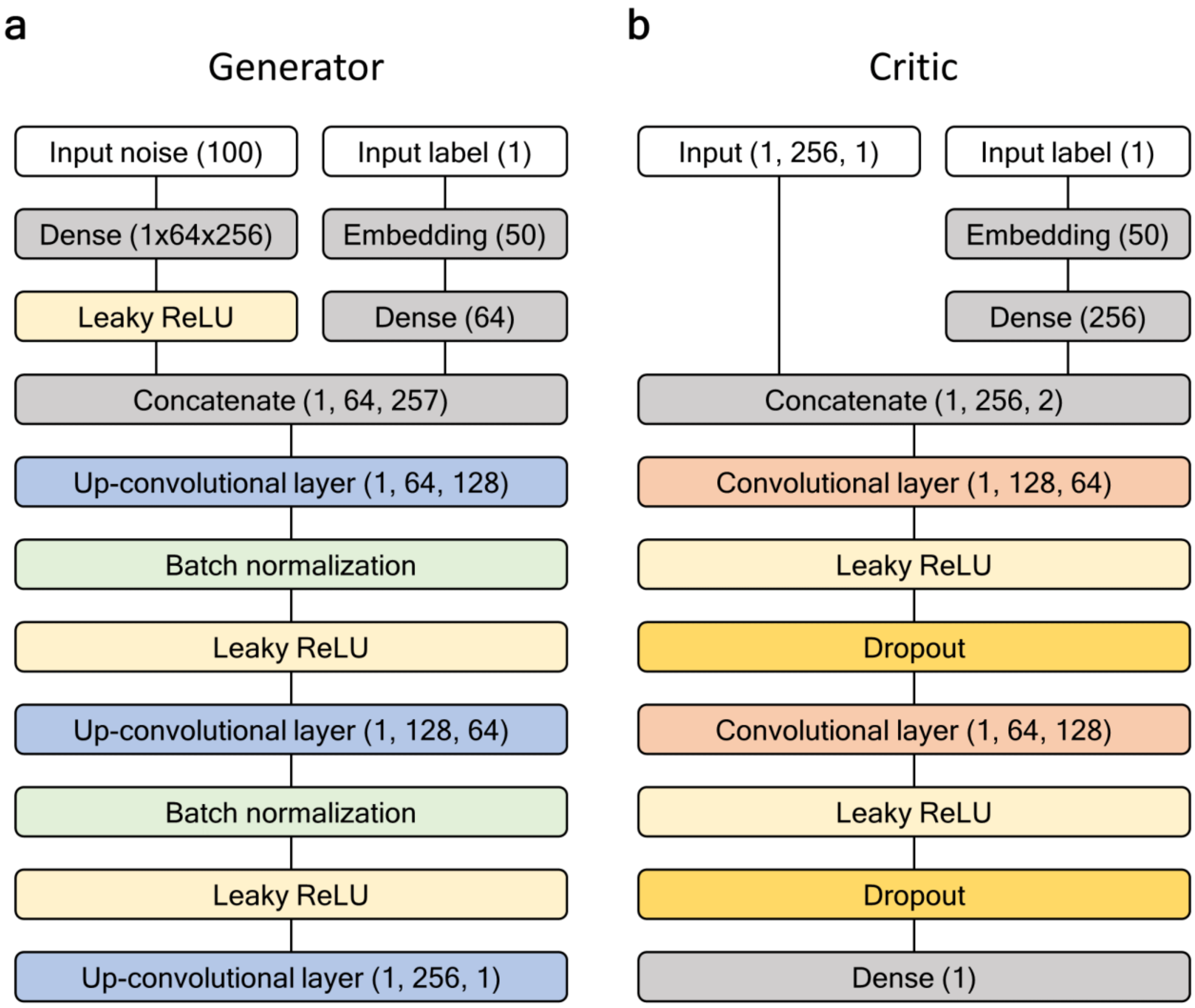

Figure S17. Conditional Wasserstein GAN architecture in DeepBioGen. **a**, A generator network generates realistic profiles from random noise. **b**, A critic network distinguishes realistic profiles from the real.

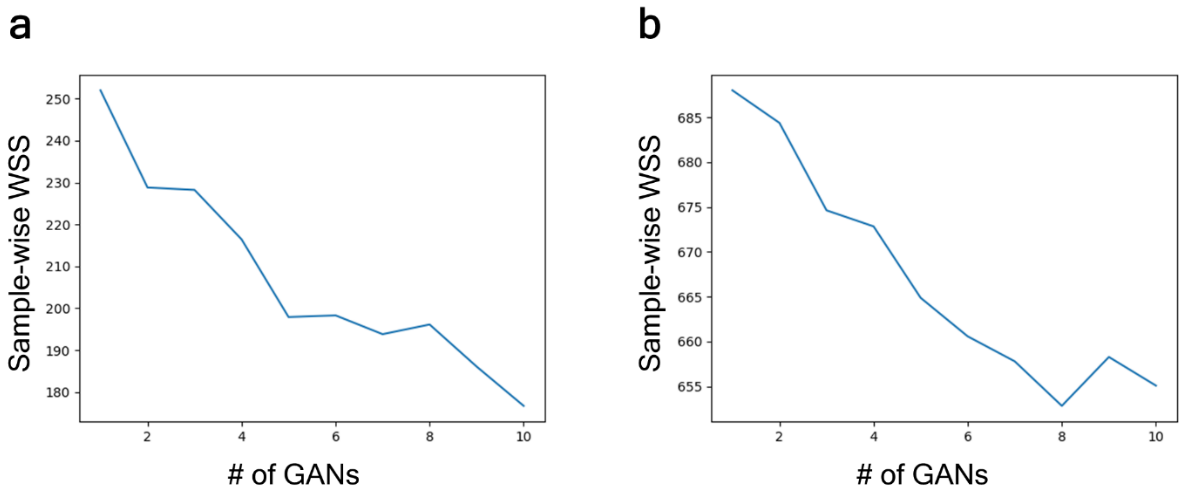

Figure S18. Sample-wise WSS by the number of GANs. **a**, RNA-seq tumor expression profiles (optimal: 5). **b**, WGS human gut microbiome marker profile (optimal: 8).

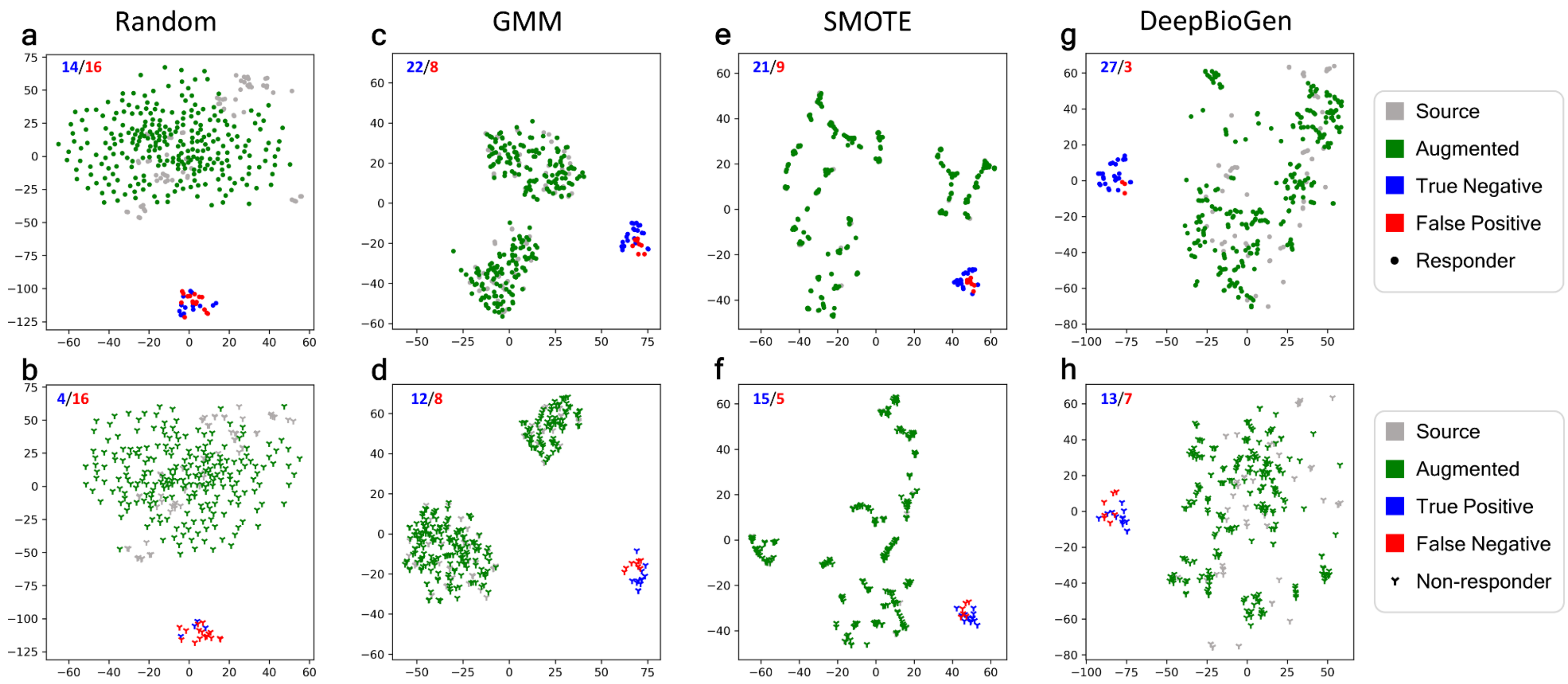

Figure S19. t-SNE visualization of anti-PD1 therapy responsiveness predictions on test data. **a-b**, predictions derived from SVM classifier based on random augmentation. **c-d**, predictions derived from SVM classifier based on GMM augmentation. **e-f**, predictions derived from SVM classifier based on SMOTE augmentation. **g-h**, predictions derived from SVM classifier based on DeepBioGen augmentation.

Table S1. Summary of sequencing data sets.

Data type		Year	# of Samples	# of class 0*	# of class 1**	Sequencing platform	# of original features	Reference
RNA-seq tumor expression profile	Source	2016	28	15	13	Illumina Hiseq 2000	25,268	Hugo et al.[1]
		2017	98	54	44	Illumina Hiseq 2000/2500	22,187	Riaz et al.[2]
	Test	2019	50	30	20	Illumina Hiseq 2500	35,678	Gide et al.[3]
WGS human gut microbiome profile	Source	2012	344	174	170	Illumina Genome Analyzer II	119,792	Qin et al.[4]
	Test	2014	96	43	53	Illumina HiSeq 2000	83,456	Karlsson et al.[5]

*Responders of anti-PD1 therapy for tumor expression profile or healthy controls for microbiome profile

**Non-responders of anti-PD1 therapy or type 2 diabetes for microbiome profile

Table S2. Hyper-parameter grid for optimizing classifiers.

Classification algorithm	Hyper-parameter	Parameter grid
SVM	Kernel	Linear and radial basis function (RBF)
	Regularization penalty *C*	2^{-4}, 2^{-3}, 2^{-2}, 2^{-1}, 2^{0}, 2^{1}, 2^{2}, and 2^{4}
	Gamma	‘Scale’ (= 1/(n_features*X.var()) and<br>‘Auto’ (=1/n_features)
RF	# of estimators	2^{7}, 2^{8}, 2^{9}, and 2^{10}
	Maximum # of features for the best split	Square root and log2 of n_features
	Split criterion	Gini impurity and information gain
NN	Hidden layers (hidden units)	3 layers (128, 64, 32),<br>4 layers (128, 64, 32, 16), and<br>5 layers (128, 64, 32, 16, 8)
	Learning rate	Constant (0.001),<br>invscaling (0.001/ pow(t, power_t) where t is time step), and<br>adaptive (keep learning rate as long as training loss is decreasing, otherwise divide the current learning rate by 5)
	Alpha (L2 penalty)	0.0001, 0.001, 0.01, and 0.1

Table S3. Modified inception scores of generated sequencing profiles varying the number of conditional Wasserstein GANs.

# of GANs	RNA-seq tumor expression profile	WGS human gut microbiome profile
1	1.0764	1.1923
2	1.0745	1.2046
3	1.0745	1.2079
4	1.0746	1.2048
5	1.0779	1.2060
6	1.0780	1.2061
7	1.0771	1.2071
8	1.0779	1.2042
9	1.0778	1.2031
10	1.0761	1.2035

Table S4. Anti-PD1 therapy responsiveness prediction accuracy of the best classifiers on unseen data.

Augmentation Method	Classifier	F1*	REC	PRE	ACC	TN	FP	FN	TP
Random	SVM	0.2	0.2	0.2	0.36	14	16	16	4
Random	RF	0.27	0.25	0.294	0.46	18	12	15	5
Random	NN	0.519	0.7	0.412	0.48	10	20	6	14
GMM	SVM	0.6	0.6	0.6	0.68	22	8	8	12
GMM	RF	0.605	0.65	0.565	0.66	20	10	7	13
GMM	NN	0.696	**0.8**	0.615	0.72	20	10	4	16
Smote	SVM	0.651	0.7	0.609	0.7	21	9	6	14
Smote	RF	0.536	0.75	0.417	0.48	9	21	5	15
Smote	NN	0.486	0.45	0.529	0.62	22	8	11	9
DeepBioGen	SVM	**0.722****	0.65	**0.812**	**0.8**	27	3	7	13
DeepBioGen	RF	0.619	0.65	0.591	0.68	21	9	7	13
DeepBioGen	NN	0.7	0.7	0.7	0.76	24	6	6	14

*F1: F-measure (Harmonic mean of precision and recall); REC: Recall; PRE: Precision; ACC: Accuracy; TN: True Negative; FP: False Positive; FN: False Negative; TP: True Positive

**The bolded value indicates the best performance across the column

Table S5. Type 2 diabetes prediction accuracy of the best classifiers on unseen data.

Augmentation Method	Classifier	F1*	REC	PRE	ACC	TN	FP	FN	TP
Random	SVM	0.511	0.434	0.622	0.542	29	14	30	23
Random	RF	0.475	0.453	0.5	0.448	19	24	29	24
Random	NN	0.521	0.472	0.581	0.521	25	18	28	25
GMM	SVM	0.707	**0.774**	0.651	0.646	21	22	12	41
GMM	RF	0.696	0.755	0.645	0.635	21	22	13	40
GMM	NN	0.706	0.679	0.735	**0.688**	30	13	17	36
Smote	SVM	0.697	0.717	0.679	0.656	25	18	15	38
Smote	RF	0.68	0.642	0.723	0.667	30	13	19	34
Smote	NN	0.7	0.66	**0.745**	**0.688**	31	12	18	35
DeepBioGen	SVM	**0.712****	0.698	0.725	**0.688**	29	14	16	37
DeepBioGen	RF	0.624	0.547	0.725	0.635	32	11	24	29
DeepBioGen	NN	0.698	0.698	0.698	0.667	27	16	16	37

*F1: F-measure (Harmonic mean of precision and recall); REC: Recall; PRE: Precision; ACC: Accuracy; TN: True Negative; FP: False Positive; FN: False Negative; TP: True Positive

**The bolded value indicates the best performance across the column

Table S6. 5-fold cross-validation on 126 tumor expression profiles (source data only).

Random Split	Clf	AUROC	STD	AUPRC	STD	ACC	STD	REC	STD	PRE	STD	F1	STD
0	SVM	0.811	0.075	0.806	0.086	0.738	0.043	0.702	0.066	0.716	0.111	0.704	0.064
1	SVM	0.791	0.034	0.725	0.126	0.699	0.052	0.685	0.164	0.664	0.103	0.657	0.104
2	SVM	0.766	0.087	0.735	0.111	0.707	0.095	0.621	0.172	0.726	0.116	0.646	0.112
3	SVM	0.712	0.100	0.658	0.181	0.620	0.078	0.636	0.122	0.597	0.129	0.598	0.075
4	SVM	0.783	0.114	0.783	0.121	0.722	0.083	0.647	0.141	0.726	0.069	0.674	0.093
5	SVM	0.707	0.041	0.642	0.165	0.603	0.020	0.577	0.114	0.594	0.146	0.557	0.037
6	SVM	0.692	0.086	0.660	0.123	0.570	0.081	0.466	0.084	0.580	0.225	0.490	0.084
7	SVM	0.749	0.106	0.680	0.185	0.626	0.095	0.579	0.083	0.628	0.214	0.583	0.100
8	SVM	0.702	0.087	0.652	0.117	0.610	0.114	0.510	0.232	0.574	0.131	0.523	0.162
9	SVM	0.673	0.068	0.622	0.201	0.603	0.100	0.447	0.128	0.557	0.201	0.491	0.149
0	RF	0.747	0.098	0.751	0.087	0.660	0.146	0.554	0.250	0.649	0.160	0.577	0.192
1	RF	0.712	0.092	0.677	0.093	0.636	0.091	0.561	0.107	0.646	0.162	0.580	0.067
2	RF	0.721	0.090	0.693	0.075	0.652	0.103	0.553	0.239	0.644	0.131	0.558	0.173
3	RF	0.693	0.047	0.638	0.100	0.643	0.026	0.606	0.177	0.641	0.147	0.591	0.072
4	RF	0.709	0.156	0.722	0.125	0.627	0.134	0.530	0.148	0.630	0.129	0.563	0.113
5	RF	0.670	0.055	0.615	0.157	0.571	0.051	0.471	0.151	0.556	0.136	0.479	0.106
6	RF	0.661	0.077	0.660	0.119	0.555	0.071	0.435	0.081	0.580	0.236	0.464	0.055
7	RF	0.733	0.099	0.698	0.152	0.658	0.085	0.537	0.114	0.729	0.241	0.582	0.091
8	RF	0.656	0.065	0.653	0.085	0.579	0.071	0.400	0.149	0.563	0.127	0.450	0.109
9	RF	0.686	0.097	0.678	0.188	0.642	0.069	0.447	0.128	0.650	0.273	0.511	0.161
0	NN	0.788	0.068	0.767	0.090	0.738	0.054	0.719	0.118	0.705	0.065	0.708	0.075
1	NN	0.718	0.083	0.621	0.168	0.674	0.049	0.640	0.124	0.637	0.125	0.630	0.102
2	NN	0.747	0.107	0.662	0.085	0.692	0.090	0.692	0.153	0.660	0.085	0.663	0.086
3	NN	0.702	0.057	0.625	0.157	0.643	0.081	0.583	0.085	0.638	0.132	0.597	0.063
4	NN	0.745	0.138	0.714	0.150	0.699	0.107	0.652	0.160	0.688	0.078	0.660	0.100
5	NN	0.689	0.094	0.627	0.198	0.587	0.053	0.608	0.071	0.562	0.142	0.565	0.060
6	NN	0.683	0.081	0.655	0.112	0.627	0.061	0.524	0.080	0.650	0.199	0.554	0.054
7	NN	0.714	0.103	0.633	0.168	0.666	0.085	0.631	0.041	0.650	0.172	0.631	0.091
8	NN	0.698	0.114	0.649	0.132	0.610	0.084	0.561	0.193	0.579	0.109	0.553	0.112
9	NN	0.653	0.050	0.572	0.180	0.659	0.060	0.558	0.131	0.602	0.127	0.577	0.126

Table S7. 5-fold cross-validation on 344 gut microbiome profiles (source data only).

Random Split	Clf	AUROC	STD	AUPRC	STD	ACC	STD	REC	STD	PRE	STD	F1	STD
0	SVM	0.727	0.022	0.699	0.043	0.683	0.026	0.707	0.035	0.672	0.060	0.686	0.029
1	SVM	0.715	0.097	0.704	0.072	0.657	0.101	0.699	0.103	0.646	0.076	0.670	0.083
2	SVM	0.731	0.049	0.715	0.074	0.686	0.059	0.702	0.053	0.683	0.095	0.688	0.054
3	SVM	0.729	0.033	0.702	0.052	0.669	0.027	0.709	0.076	0.655	0.048	0.676	0.023
4	SVM	0.726	0.046	0.713	0.057	0.678	0.056	0.697	0.072	0.668	0.067	0.680	0.057
5	SVM	0.728	0.044	0.699	0.074	0.683	0.039	0.688	0.041	0.676	0.080	0.679	0.051
6	SVM	0.731	0.097	0.719	0.106	0.677	0.098	0.728	0.102	0.663	0.099	0.690	0.087
7	SVM	0.726	0.041	0.703	0.045	0.672	0.042	0.731	0.097	0.654	0.036	0.685	0.040
8	SVM	0.750	0.032	0.737	0.066	0.694	0.036	0.693	0.078	0.697	0.078	0.689	0.045
9	SVM	0.745	0.034	0.741	0.054	0.648	0.051	0.663	0.082	0.647	0.105	0.645	0.062
0	RF	0.726	0.014	0.701	0.041	0.645	0.036	0.602	0.027	0.659	0.071	0.626	0.022
1	RF	0.712	0.113	0.698	0.082	0.660	0.104	0.623	0.103	0.675	0.092	0.646	0.093
2	RF	0.710	0.026	0.694	0.050	0.657	0.030	0.621	0.075	0.664	0.059	0.638	0.049
3	RF	0.728	0.049	0.707	0.059	0.666	0.076	0.616	0.094	0.681	0.115	0.642	0.090
4	RF	0.728	0.028	0.692	0.062	0.657	0.034	0.616	0.054	0.668	0.032	0.639	0.026
5	RF	0.721	0.046	0.691	0.071	0.640	0.039	0.624	0.073	0.647	0.091	0.628	0.044
6	RF	0.720	0.102	0.716	0.113	0.660	0.097	0.673	0.117	0.650	0.090	0.660	0.097
7	RF	0.728	0.029	0.705	0.047	0.651	0.036	0.666	0.101	0.647	0.030	0.650	0.043
8	RF	0.709	0.055	0.703	0.106	0.654	0.018	0.636	0.082	0.664	0.069	0.642	0.032
9	RF	0.721	0.050	0.708	0.066	0.642	0.045	0.611	0.064	0.658	0.095	0.624	0.031
0	NN	0.726	0.025	0.698	0.035	0.651	0.040	0.640	0.061	0.651	0.080	0.642	0.054
1	NN	0.709	0.086	0.704	0.065	0.678	0.081	0.674	0.068	0.682	0.070	0.676	0.061
2	NN	0.711	0.058	0.687	0.099	0.663	0.056	0.684	0.077	0.658	0.095	0.665	0.061
3	NN	0.714	0.044	0.694	0.065	0.654	0.063	0.641	0.122	0.654	0.065	0.641	0.077
4	NN	0.727	0.054	0.712	0.078	0.654	0.051	0.628	0.070	0.659	0.049	0.641	0.050
5	NN	0.727	0.043	0.692	0.081	0.660	0.031	0.644	0.043	0.664	0.087	0.649	0.042
6	NN	0.728	0.096	0.711	0.108	0.662	0.101	0.686	0.097	0.656	0.099	0.668	0.090
7	NN	0.725	0.031	0.697	0.044	0.646	0.051	0.657	0.142	0.642	0.029	0.639	0.076
8	NN	0.754	0.041	0.745	0.052	0.671	0.054	0.631	0.104	0.680	0.086	0.650	0.077
9	NN	0.738	0.052	0.746	0.041	0.663	0.055	0.641	0.102	0.674	0.064	0.648	0.036

Table S8. 5-fold cross-validation on 176 tumor expression profiles (source + test data).

Random Split	Clf	AUROC	STD	AUPRC	STD	ACC	STD	REC	STD	PRE	STD	F1	STD
0	SVM	0.738	0.106	0.705	0.089	0.682	0.087	0.663	0.099	0.639	0.083	0.646	0.073
1	SVM	0.747	0.095	0.690	0.138	0.659	0.090	0.598	0.123	0.623	0.173	0.599	0.124
2	SVM	0.691	0.087	0.651	0.081	0.626	0.095	0.620	0.211	0.584	0.125	0.579	0.113
3	SVM	0.734	0.108	0.685	0.100	0.665	0.092	0.631	0.154	0.637	0.105	0.617	0.097
4	SVM	0.709	0.056	0.654	0.008	0.630	0.065	0.590	0.090	0.586	0.108	0.579	0.078
5	SVM	0.745	0.098	0.641	0.170	0.710	0.098	0.604	0.180	0.685	0.146	0.633	0.145
6	SVM	0.744	0.086	0.670	0.147	0.705	0.069	0.607	0.087	0.690	0.154	0.641	0.103
7	SVM	0.734	0.083	0.645	0.187	0.653	0.082	0.582	0.123	0.642	0.209	0.586	0.100
8	SVM	0.723	0.102	0.655	0.155	0.636	0.075	0.536	0.161	0.599	0.178	0.549	0.142
9	SVM	0.720	0.052	0.685	0.096	0.647	0.031	0.549	0.137	0.619	0.078	0.567	0.073
0	RF	0.709	0.111	0.704	0.092	0.631	0.147	0.567	0.223	0.576	0.105	0.559	0.168
1	RF	0.717	0.124	0.671	0.188	0.630	0.102	0.463	0.073	0.643	0.240	0.524	0.125
2	RF	0.640	0.102	0.604	0.116	0.574	0.070	0.446	0.192	0.524	0.133	0.459	0.126
3	RF	0.709	0.113	0.688	0.119	0.636	0.057	0.516	0.103	0.610	0.126	0.549	0.076
4	RF	0.675	0.065	0.617	0.076	0.557	0.086	0.477	0.122	0.511	0.132	0.480	0.089
5	RF	0.706	0.098	0.656	0.154	0.648	0.092	0.489	0.128	0.621	0.169	0.543	0.141
6	RF	0.728	0.088	0.671	0.163	0.648	0.065	0.497	0.127	0.641	0.138	0.546	0.096
7	RF	0.702	0.087	0.638	0.174	0.636	0.085	0.537	0.128	0.629	0.196	0.555	0.080
8	RF	0.703	0.067	0.643	0.109	0.665	0.085	0.559	0.091	0.646	0.139	0.591	0.088
9	RF	0.702	0.023	0.650	0.054	0.637	0.059	0.471	0.196	0.624	0.123	0.510	0.137
0	NN	0.732	0.130	0.695	0.125	0.694	0.101	0.707	0.163	0.643	0.120	0.663	0.114
1	NN	0.717	0.108	0.638	0.197	0.670	0.093	0.623	0.120	0.615	0.179	0.614	0.140
2	NN	0.638	0.094	0.571	0.093	0.626	0.080	0.577	0.159	0.592	0.120	0.566	0.090
3	NN	0.717	0.080	0.630	0.145	0.664	0.080	0.600	0.120	0.628	0.074	0.606	0.081
4	NN	0.693	0.071	0.593	0.082	0.618	0.099	0.611	0.157	0.568	0.110	0.575	0.118
5	NN	0.720	0.119	0.646	0.181	0.711	0.122	0.562	0.197	0.702	0.200	0.615	0.185
6	NN	0.731	0.091	0.648	0.153	0.688	0.087	0.637	0.096	0.661	0.157	0.641	0.103
7	NN	0.738	0.092	0.637	0.164	0.687	0.033	0.640	0.091	0.659	0.127	0.633	0.032
8	NN	0.726	0.099	0.632	0.183	0.653	0.073	0.563	0.133	0.608	0.153	0.577	0.122
9	NN	0.705	0.043	0.636	0.056	0.631	0.042	0.501	0.121	0.616	0.090	0.534	0.065

Table S9. 5-fold cross-validation on 440 gut microbiome profiles (source + test data).

Random Split	Clf	AUROC	STD	AUPRC	STD	ACC	STD	REC	STD	PRE	STD	F1	STD
0	SVM	0.723	0.057	0.733	0.061	0.636	0.069	0.691	0.082	0.628	0.058	0.657	0.066
1	SVM	0.740	0.029	0.755	0.053	0.679	0.035	0.751	0.057	0.662	0.041	0.702	0.036
2	SVM	0.727	0.042	0.739	0.081	0.657	0.049	0.720	0.052	0.647	0.075	0.678	0.048
3	SVM	0.706	0.056	0.714	0.061	0.652	0.050	0.683	0.081	0.645	0.055	0.663	0.067
4	SVM	0.728	0.040	0.726	0.050	0.654	0.029	0.703	0.082	0.653	0.065	0.671	0.030
5	SVM	0.736	0.045	0.723	0.061	0.682	0.016	0.690	0.056	0.684	0.039	0.685	0.034
6	SVM	0.723	0.059	0.716	0.095	0.654	0.041	0.700	0.026	0.649	0.090	0.670	0.048
7	SVM	0.727	0.023	0.719	0.056	0.661	0.054	0.683	0.048	0.662	0.031	0.672	0.037
8	SVM	0.739	0.023	0.749	0.036	0.664	0.034	0.720	0.053	0.656	0.063	0.683	0.036
9	SVM	0.748	0.046	0.751	0.083	0.687	0.076	0.734	0.067	0.678	0.105	0.701	0.079
0	RF	0.723	0.063	0.732	0.055	0.632	0.067	0.617	0.085	0.642	0.071	0.628	0.073
1	RF	0.717	0.033	0.704	0.088	0.666	0.028	0.657	0.046	0.676	0.047	0.664	0.028
2	RF	0.713	0.029	0.711	0.075	0.639	0.024	0.616	0.082	0.663	0.075	0.630	0.033
3	RF	0.727	0.048	0.726	0.041	0.666	0.058	0.642	0.095	0.674	0.069	0.657	0.083
4	RF	0.708	0.057	0.704	0.082	0.634	0.047	0.613	0.093	0.655	0.081	0.626	0.049
5	RF	0.730	0.065	0.718	0.080	0.673	0.045	0.655	0.067	0.685	0.058	0.668	0.054
6	RF	0.715	0.038	0.705	0.085	0.659	0.028	0.654	0.041	0.666	0.091	0.657	0.051
7	RF	0.731	0.028	0.713	0.064	0.664	0.045	0.646	0.035	0.676	0.050	0.660	0.039
8	RF	0.729	0.037	0.709	0.074	0.652	0.033	0.659	0.068	0.662	0.057	0.656	0.035
9	RF	0.739	0.038	0.731	0.057	0.664	0.041	0.639	0.078	0.679	0.093	0.654	0.062
0	NN	0.728	0.071	0.730	0.084	0.652	0.071	0.661	0.097	0.652	0.068	0.655	0.079
1	NN	0.735	0.033	0.727	0.059	0.702	0.028	0.736	0.050	0.693	0.038	0.713	0.034
2	NN	0.723	0.041	0.735	0.088	0.648	0.032	0.638	0.062	0.662	0.063	0.646	0.028
3	NN	0.701	0.067	0.711	0.069	0.643	0.070	0.646	0.119	0.641	0.082	0.642	0.098
4	NN	0.736	0.046	0.756	0.032	0.691	0.042	0.719	0.093	0.692	0.029	0.700	0.031
5	NN	0.733	0.045	0.715	0.059	0.675	0.024	0.650	0.055	0.690	0.038	0.668	0.036
6	NN	0.719	0.049	0.712	0.089	0.650	0.053	0.667	0.025	0.656	0.103	0.657	0.053
7	NN	0.726	0.025	0.717	0.052	0.666	0.039	0.699	0.051	0.660	0.035	0.678	0.039
8	NN	0.736	0.029	0.725	0.046	0.655	0.032	0.676	0.046	0.660	0.055	0.664	0.019
9	NN	0.727	0.035	0.723	0.080	0.673	0.029	0.689	0.029	0.673	0.074	0.678	0.040

References

1 Hugo, W. *et al.* Genomic and transcriptomic features of response to anti-PD-1 therapy in metastatic melanoma. *Cell* **165**, 35-44 (2016).

2 Riaz, N. *et al.* Tumor and microenvironment evolution during immunotherapy with nivolumab. *Cell* **171**, 934-949. e916 (2017).

3 Gide, T. N. *et al.* Distinct immune cell populations define response to anti-PD-1 monotherapy and anti-PD-1/anti-CTLA-4 combined therapy. *Cancer cell* **35**, 238-255. e236 (2019).

4 Qin, J. *et al.* A metagenome-wide association study of gut microbiota in type 2 diabetes. *Nature* **490**, 55-60 (2012).

5 Karlsson, F. H. *et al.* Gut metagenome in European women with normal, impaired and diabetic glucose control. *Nature* **498**, 99-103 (2013).
